# Healthcare workers’ perspectives on patient bypassing of Primary Health Care facilities in Mpumalanga province, South Africa

**DOI:** 10.4102/phcfm.v18i1.5368

**Published:** 2026-05-11

**Authors:** Thobelani N. Majola, Ntombifuthi Blose, Mashudu Mthethwa, Zinhle Mtwane, Algernon M. Africa, Matome S. Mokganya, James M. Burnett, Noluthando Ndlovu, Ashnie Padarath

**Affiliations:** 1 Department of Research and Implementation Science, Health Systems Trust, Durban, South Africa; 2 Centre for Epidemic Response and Innovation, School for Data Science and Computational Thinking, South African Centre for Epidemiological Modelling and Analysis (SACEMA), Stellenbosch University, Stellenbosch, South Africa; 3 School of Health Systems and Public Health, Faculty of Health Sciences, University of Pretoria, Pretoria, South Africa

**Keywords:** bypassing, Primary Health Care, non-referred, patients, healthcare workers, Mpumalanga

## Abstract

**Background:**

South Africa’s Referral Policy and Implementation Guidelines aim to ensure continuity of care, manage patient flow, and improve health system efficiency. However, in Mpumalanga province, a growing number of patients bypass Primary Health Care (PHC) clinics and seek hospital services without formal referral. This trend undermines the structured referral pathway and strains healthcare resources.

**Aim:**

This study sought to identify the key factors that influence patients to bypass PHC facilities in Mpumalanga province.

**Setting:**

The study was conducted across the three districts in Mpumalanga province.

**Methods:**

A qualitative exploratory design was employed to better understand the factors influencing patients to bypass PHC clinics from the perspective of healthcare workers (HCWs). Using purposive sampling techniques, key informant interviews were conducted with 20 HCWs from 10 selected hospitals. Thematic analysis of the qualitative data was conducted using an inductive approach, with NVivo^®^ software facilitating systematic coding, theme development and data organisation.

**Results:**

Key factors that influenced patients to bypass included a lack of adherence to the referral system, perceptions of poor quality at PHC facilities, long waiting times, negative HCWs’ attitudes and concerns pertaining to stigma and confidentiality.

**Conclusion:**

Insights gained from this study contribute to the understanding of factors that lead patients to bypass PHC facilities.

**Contribution:**

By understanding the underlying reasons for such bypassing, policymakers and healthcare providers can develop targeted interventions to enhance the quality and utilisation of PHC services. Moreover, addressing these challenges is crucial for achieving equitable healthcare access.

## Introduction

The Primary Health Care (PHC) approach as formulated by the World Health Organization in the Alma-Ata Declaration, is a philosophy that guides the principles and strategies for organising health systems.^1,2^ This is centred on and represents the notion of health as a human right, with health systems seen as the vehicle to deliver this right equitably. The PHC approach consists of three main elements, namely multisectoral policy and action; empowered people and communities; as well as primary care and essential public health functions as the core of integrated services.^1^ South Africa formally adopted the PHC approach as the foundation of the health system in 1994, focusing on universal access and decentralisation of services, which catalysed major system changes including the amalgamation of 14 previously racially divided health departments into one national health system, and the removal of user fees for PHC facilities.^2^ In South Africa, PHC clinics and Community Health Centres (CHCs) constitute the first formal institutional point of contact for people seeking health services. This is followed by district, regional, tertiary and central hospitals, depending on the referral and condition of each patient.^3^ The country’s referral system forms part of the comprehensive healthcare service delivery platform to manage healthcare needs by referring patients from an initiating facility to an organisation or service that can provide the appropriate level of care.^3^ For the referral system to be effective, all levels of health service delivery should function optimally, with patients accessing PHC facilities first, and following clinical examination, being referred to higher levels of care if required. Although the referral system was outlined in accordance with the Referral Policy for South African Health Services in 2020, its implementation continues to face several challenges. One such challenge is rooted in low levels of compliance with the referral policy among patients. It has been observed that although the first place of contact with the public healthcare sector is the PHC clinic or CHC, there is a concerning trend of patients bypassing PHC facilities and accessing hospitals directly, that is, without a referral letter. This non-referred bypassing occurs when patients deliberately access hospitals without first engaging at the PHC level.^4^ It is therefore critical to determine the factors influencing patients’ decision to bypass PHC facilities, particularly in Mpumalanga province, where approximately 54% of new patients report to public hospitals without being referred, which ranks it as the province with the third-highest prevalence of this trend nationally.^5^

### Factors leading patients to bypass Primary Health Care facilities

According to Koce et al.^6^ the healthcare referral system is designed to ensure that patients receive care at the appropriate level. However, some patients do not adhere to the referral system. The various factors that influence a patient’s decision to bypass the PHC level of care and present themselves at the hospital without a referral letter can be divided into two main categories, namely individual-level factors and health system factors.

### Individual-level factors

Evidence from several studies shows that local healthcare facilities are not frequently utilised and/or are bypassed by individuals in favour of secondary facilities, even when care could be managed at the PHC level.^6,7,8^ Various individual factors contribute to non-referred patients’ decision to seek care at hospitals. The absence of medical officers in certain PHC facilities deters patients from presenting to those facilities, despite the availability of other healthcare workers (HCWs) such as nurses.^6^ Moreover, the idea of being attended by a medical doctor at the hospital contributes to the cumulative number of patients who bypassed PHC facilities.^6^ The decision to utilise PHCs is often determined by the individual’s perceptions of the quality of healthcare services.^9^ Certain patients tend to avoid PHC facilities based on the belief that healthcare services are not delivered optimally at this level. A South African study conducted in Pretoria revealed that the common reason for patients bypassing the nearest clinic was the perception that they received better service and advanced care at the hospitals.^8^ Similarly, a study conducted in Uganda found that patients were bypassing PHC facilities because of poor quality of services experienced at this level.^10^ Patients’ satisfaction with the services received at PHC facilities influenced their decision to either return to those facilities or to seek medical services elsewhere. Patients’ perception of the severity of their condition was another factor contributing to them seeking care from either a PHC facility or a hospital.^8^ South Africa has a high burden of communicable diseases such as Human Immunodeficiency Virus (HIV) and tuberculosis (TB), as well as non-communicable conditions such as hypertension and cardiovascular diseases, diabetes, cancer and mental illness.^8^ Some patients receiving treatment choose to access facilities outside their communities to avoid judgement, stigma and discrimination associated with their conditions. Their fear of judgement and stigma associated with being infected with certain diseases has led them to opt for healthcare facilities located outside the community. This is substantiated by a study conducted in KwaZulu-Natal province, which found that most people who are on TB and/or HIV treatment chose to seek care from facilities that were far from where they lived, based on concerns that community members would judge them because of their illnesses.^11^ The differentiation of consultation rooms for patients with chronic illnesses such as HIV was perceived as discriminatory and facilitating stigma, while the lack of privacy and confidentiality that some patients encountered at a PHC facility deterred them from returning.^8^

### Health system factors

The accessibility of PHC facilities can result in patients seeking care from a hospital located closer to their household. The distance to a facility and geographical access are key considerations in facility selection, particularly for individuals with limited mobility, such as the elderly and people living with a disability.^12^ Koce et al.^6^ observed in their study that patients self-referred themselves to hospitals if PHC facilities were located far from their residence. The availability of transport and associated costs also play a role in a patient’s decision to select a facility for health services. The South African healthcare system faces several challenges, including shortages of human resources and medicines, with PHC facilities being markedly affected in this regard.^13^ Staff shortages have a negative impact on service delivery and the provision of quality healthcare. Specifically, such shortages cause long waiting times for patients, creating patient dissatisfaction, which leads them to seek care from the hospital.^6,13^ Several studies have shown that medication stock-outs play a role in patients deliberately bypassing PHC facilities and obtaining care directly from hospitals.^8,11^ In addition, the Ritshidze^14^ *Mpumalanga State of Health Report 2021* revealed that 10.7% of patients left PHC facilities without the medication they needed; 39.5% of facilities had shortages of contraceptives, while 39.5% of facilities had shortages in antiretroviral therapy. Some patients self-refer to hospital emergency departments on weekends and in the evenings because of the limited operational hours of PHC facilities.^8^ According to this *Mpumalanga State of Health Report*, only 48.7% of patients thought that the staff were always friendly and professional in most PHC facilities.^14^ A study on barriers to quality healthcare in Mpumalanga province found that some HCWs demonstrated negative attitudes and a lack of empathy towards patients, which contributed to the provision of poor-quality care.^15^ This is supported by a study conducted in Limpopo province, which found that the attitude of HCWs in PHC facilities contributed to patients’ preference for hospitals.^9^

The approved 2020 Referral Policy for South African Health Services and Referral Implementation Guidelines clearly stipulate the protocols for care provided at different levels of the healthcare system.^3^ Knowledge of the referral policy is deemed necessary for the system to work effectively, while a lack of knowledge about the referral system has contributed to certain patients seeking care at the hospital without a referral letter. Findings from a study conducted in Pretoria suggest that 48.8% of patients had limited knowledge about the referral process and the nature of medical care offered at different levels of the health system.^8^ The study also found that while 11.1% of patients had such knowledge, they did not fully understand the system.^8^ Similarly, a study undertaken in KwaZulu-Natal found that 53.6% of self-referred patients had knowledge of the referral procedure.^11^ While some patients may be aware of the referral system, there remains a knowledge gap, as a significant proportion of patients do not fully understand the referral system. This lack of knowledge and understanding is undoubtedly resulting in many patients bypassing PHC facilities.

## Research methods and design

### Study design

The study employed a qualitative exploratory design to understand HCWs’ perspectives on factors influencing patients’ decisions to bypass PHC facilities. An exploratory qualitative design is appropriate when investigating a relatively underexplored phenomenon or seeking to generate insights into contextual drivers and service utilisation patterns.^16^ The design facilitated an in-depth examination of HCWs’ and managers’ viewpoints, allowing for rich insights into perceived systemic, organisational, and behavioural determinants of bypassing. Given the limited empirical evidence on patient bypass behaviour within the South African health system, the exploratory design enabled the identification of emergent themes and nuanced interpretations of healthcare system dynamics that may inform policy and service delivery improvements.

### Setting

Mpumalanga province consists of three districts, namely Ehlanzeni, Nkangala and Gert Sibande,^17^ and has 33 public hospitals and 287 public health clinics. The Ehlanzeni District Municipality is located in the north-east of Mpumalanga province.^18^ The district comprises four local municipalities, with an estimated population of 1 853 931.^19^ Ehlanzeni has 141 healthcare facilities made up of 110 clinics, 15 CHCs, 11 district, regional and tertiary hospitals, and five ‘other’ hospitals.^20^ Gert Sibande District Municipality is located in Mpumalanga province and borders eSwatini.^18^ The district comprises seven local municipalities with an estimated population of 1 283 719.^19^ Gert Sibande had 90 healthcare facilities in 2017, made up of 57 PHC clinics, 19 CHCs, nine district and regional hospitals, and five ‘other’ hospitals.^21^ Nkangala District Municipality consists of an estimated population of 1 677 408.^19^ It is the smallest district of the three in the province, consisting of six local municipalities. In total, Nkangala has 117 healthcare facilities, made up of 74 clinics, 22 CHCs, eight district and tertiary hospitals and 13 ‘other’ hospitals.^22^ A total of 10 hospitals were selected across the three districts. Hospitals were selected using District Health Information System (DHIS) data, based on the historical average outpatient headcount for ‘Outpatient Department (OPD) New Client Not Referred’ across the 2019/20–2022/23 financial years. Because the historical average was used as a benchmark, the computation therefore excluded the current 2023/24 financial year. The hospitals were then classified as ‘High’ if the historical average was lower than the metric of the 2023/24 OPD New Client Not Referred headcount, or ‘Low’ if it was equal or higher than this headcount. Hospitals were then stratified by rural and urban geographical categories per district municipality. This allowed for the selection of hospitals to be grouped within each geographical type rather than between them. Hospitals were identified, with the first 10 hospitals that provided support letters being selected, four of which were in rural settings and six in urban areas across all three districts. Eight of the hospitals were district facilities, one was a regional hospital, and one was a tertiary hospital.

### Study population and sampling strategy

The study targeted HCWs and managers employed in the 10 selected hospitals, as they play a critical role in providing health-related guidance to patients. Moreover, HCWs frequently interact with patients, making their perspectives essential to the study. A purposive sampling approach was adopted to select HCWs in Mpumalanga province. This entailed selecting HCWs deemed relevant to achieve the research objectives.^23^ A total of 20 HCWs and managers were selected from 10 hospitals in Mpumalanga province.

### Data collection

The recruitment and facilitation of interviews occurred between 04 and 22 November 2024. Key informant interviews were conducted with 20 HCWs and managers within the premises of the 10 hospitals. A key informant interview guide was designed, consisting of questions relating to HCWs’ perspectives of the reasons patients bypass PHC facilities; patients’ knowledge about the healthcare system; as well as recommendations to improve the utilisation of PHC services in Mpumalanga province.

### Data analysis

Thematic analysis of data was undertaken using NVivo^®^ data analysis software. The qualitative coding method was used to identify and extract common ideas, patterns or themes from the discussions. Thematic analysis is well suited to interpreting qualitative data, as it is a flexible and versatile method that can be applied using either inductive or deductive approaches.^24^ An inductive approach was used, which involved reading through the data and identifying codes, patterns, and themes as they emerged. This was guided by Braun and Clarke’s^25^ six steps of thematic analysis, which entail familiarisation with the data, coding, generation of themes, reviewing themes, defining and naming themes, and producing the final report. In this study on HCWs’ perspectives of patients’ bypassing of PHC facilities, familiarisation involved repeated reading of transcripts to gain an in-depth understanding of participants’ accounts. Initial coding was conducted inductively to capture meaningful units of data related to perceived drivers of bypass behaviour. Codes were then grouped into potential themes, which were reviewed and refined to ensure coherence and clear distinctions between themes. Themes were subsequently defined and named to reflect their analytic essence, exploring factors leading non-referred patients to seek care directly at hospitals; patients’ knowledge of the referral system; and health systems strategies related to referral and service utilisation. The final analytic narrative was produced with supporting quotations to enhance transparency and depth of interpretation regarding the factors influencing patients’ decisions to bypass primary care facilities. To ensure that the data analysis was robust and open to additional factors so as not to limit participants, an open factor was added during the analysis process, namely ‘Other’.

Ensuring trustworthiness is essential in qualitative research to enhance the rigour and credibility of findings.^26^ Trustworthiness was strengthened through strategies addressing credibility, dependability, confirmability, and transferability. Credibility was enhanced through iterative engagement with the data, persistent observation during analysis, and the use of direct quotations to ground themes in HCWs’ perspectives. As noted by Korstjens and Moser,^27^ persistent observation contributes to the credibility of qualitative findings by ensuring depth and accuracy of interpretation. Dependability was supported by a transparent description of the analytic process guided by the six-step thematic analysis framework,^25^ ensuring systematic coding and theme development. Confirmability was strengthened through the maintenance of an audit trail and reflexive notes to minimise researcher bias. Although transferability was not the primary intention of this qualitative study, as it sought to explore context-specific perspectives rather than generate statistically generalisable findings, it was supported through the provision of detailed descriptions of the study setting, participants’ roles, and health system context, enabling readers to determine the applicability of findings to similar settings.

### Ethical considerations

The study received approval from an Independent Ethics Committee, Pharma-Ethics (Ethics Reference Number: 240726606). Authorisation to conduct the study was granted by the National Department of Health and the relevant Provincial Health Research Ethics Committee under the National Health Research Database reference number MP_202410_004. Support letters were also provided by the Chief Executive Officers of the selected hospitals. Written informed consent was obtained from all participants beforehand.

## Results

The results of the study are presented first by outlining participant demographics, followed by findings from interviews with HCWs and managers.

### Socio-demographic characteristics of healthcare workers and managers

The socio-demographic characteristics of HCWs and managers interviewed across the three districts are summarised in Table 1. Overall, eight of those interviewed were between 31 and 40 years of age, four were aged 41–50 years, three were aged 51–60 years, and five were between 61 and 70 years old. The majority of participants were female (*n* = 14). Regarding educational qualifications, 16 of the participants held either a degree, diploma, or postgraduate diploma in nursing. Regarding occupational categories, 11 of the key informants were nurses, including two Assistant Nurses, one Enrolled Nurse, two Registered Nurses and six Professional Nurses. In addition, five Operational Managers and four Medical Doctors were interviewed.

**TABLE 1:** Socio-demographic characteristics of healthcare workers and managers.

Characteristic	Category	Districts						Total	
		Ehlanzeni		Gert Sibande		Nkangala			
		*n*	%	*n*	%	*n*	%	*n*	%
Age (years)	31–40	5	25	2	10	1	5	8	40
	41–50	2	10	0	0	2	10	4	20
	51–60	2	10	0	0	1	5	3	15
	61–70	1	5	2	10	2	10	5	25
Sex	Female	7	35	3	15	4	20	14	70
	Male	3	15	1	5	2	10	6	30
Educational attainment	Master’s degree in Medicine	0	0	0	0	1	5	1	5
	Bachelor of Medicine	1	5	1	5	1	5	3	15
	Postgraduate Diploma in Nursing	0	0	0	0	1	5	1	5
	Degree in Nursing	3	15	0	0	1	5	4	20
	Diploma in Nursing	6	30	3	15	2	10	11	55
Occupation	Operational Managers	1	5	0	0	4	20	5	25
	Medical Doctors	1	5	1	5	2	10	4	20
	Professional Nurses	6	30	0	0	0	0	6	30
	Registered Nurses	0	0	2	10	0	0	2	10
	Enrolled Nurses	0	0	1	5	0	0	1	5
	Assistant Nurses	2	10	0	0	0	0	2	10

### Findings from healthcare workers and managers

Various themes were identified from the study’s findings. These results relate to HCWs’ perspectives and experiences on factors that are leading patients to bypass PHC facilities in Mpumalanga province.

### Knowledge of the referral system

There seemed to be limited information about the referral system among some patients. The HCWs reported that certain patients sought care directly at hospitals, unaware that clinics are intended to be the first point of contact within the public healthcare system. Of the 20 HCWs interviewed, six reported that patients lacked knowledge of the referral process. Interviews further revealed that when HCWs asked patients why they bypassed clinics, their responses often reflected this lack of information. For instance, some young mothers brought their children to the casualty without first visiting a clinic:

‘Some don’t know and I think my encounters with the ones that didn’t know were young mothers, some young mothers would bring the child straight to casualty without starting at the clinic.’ (Participant 4, Medical Doctor, Male)

‘There isn’t enough awareness. Patients just seek care wherever they want without understanding the system.’ (Participant 2, Operational Manager, Male)

Despite limited knowledge among some patients, there was evidence that some participants demonstrated adequate awareness of the referral system. Interviews indicated that while most patients were familiar with referral pathways, they nonetheless sought care directly from hospitals. Of the 20 HCWs interviewed, 14 confirmed that patients generally possessed knowledge of the referral process. However, patients reported preferring hospitals over clinics, citing perceived delays and inefficiencies at PHC facilities:

‘According to my assessment, I think patients know about the referral system. They will tell you, I didn’t start at the clinic because they waste my time.’ (Participant 7, Professional Nurse, Female)

‘Yes, they’re aware because each and every morning, I give health talk. I give health talk every day in the morning and we have a piece of paper in the triage room.’ (Participant 1, Assistant Nurse, Female)

Although the findings suggest that most patients were aware of the referral system, adherence was not consistent. Interviews revealed that some patients, despite having knowledge of the system, chose to present directly at hospitals, knowing that they would still receive care even without following the appropriate referral pathways. The HCWs further indicated that certain patients demonstrated a lack of respect for established protocols, with some displaying a sense of entitlement to public healthcare services, which contributed to non-adherence to the referral system:

‘Even though they know, it’s just attitude. Some patients don’t respect us healthcare workers and they don’t follow the proper structures. If they want to go to the hospital they will come because they know we will assist them, there is no way we can turn them away, I think they take advantage of that.’ (Participant 10, Medical Doctor, Female)

‘In some instances, you also find patients that are aware of the referral pathway, but they feel that clinics are not helping them, hence they opted to come to the hospital. Some don’t care about the system.’ (Participant 5, Operational Manager, Female)

### Perception of services

The perception that hospitals offer better quality of care appears to significantly influence the growing trend of non-referred patients presenting directly to hospital facilities. The HCWs reported that some patients expressed a preference for hospital-based consultations, primarily because of the expectation of being attended to by a medical doctor. This preference was rooted in a belief that medical doctors, as opposed to nurses, were likely to provide accurate diagnoses and informed treatment decisions. Consequently, patients frequently sought hospital care for conditions that were non-urgent or could have been managed at the primary care level. In addition, the availability of essential resources such as pharmaceuticals and diagnostic equipment (x-rays) was identified as a motivating factor in patients’ decision-making processes:

‘Most of the patients feel like doctors will treat you better than nurses, they feel like doctors are good. If they touch you with the stethoscope, then you’d be healed.’ (Participant 4, Medical Doctor, Male)

‘Patients prefer hospitals because they know they can get x-rays quickly, if they are coughing or the doctors need to do more examination, they will be examined and x-rays will be done.’ (Participant 12, Operational Manager, Female)

The HCWs reported that patients frequently cited long queues and extended waiting times as reasons for bypassing clinics in favour of hospitals. They explained that patients compared waiting times at clinics unfavourably with those at hospitals, often believing that hospital services were more efficient and responsive. These perceptions of prolonged delays and inefficiency at PHC facilities appeared to influence patients’ decisions to seek care directly at hospitals, reinforcing bypass behaviour even in the absence of formal referral:

‘Long lines at the clinics; they complain about lines being longer than here.’ (Participant 10, Medical Doctor, Female)

‘They avoid the queueing … they feel that they queue for longer and the service is slow.’ (Participant 5, Operational Manager, Female)

As the first point of contact, clinics face several service delivery challenges that shape patients’ perceptions of care. During interviews, HCWs identified staff shortages as a major constraint contributing to underutilisation of PHC services and negative perceptions of service quality. In many facilities, the patient-to-provider ratio was reported to be disproportionately high, resulting in prolonged waiting times and extended hours spent at clinics. Inadequate infrastructure was perceived to restrict the number of patients who could be attended to simultaneously, further exacerbating waiting times and contributing to overcrowding. These structural limitations reinforced perceptions of inefficiency and poor service organisation, ultimately influencing some patients’ decisions to bypass PHC facilities in favour of hospitals:

‘At the clinic, we don’t have enough consulting rooms, not enough equipment and the staff is also not enough to cater for the number of patients that come to the clinic.’ (Participant 11, Registered Nurse, Female)

‘And then secondly, they [*the patients*] will tell you that clinics are always full, there are always long queues.’ (Participant 5, Operational Manager, Female)

### Medicine availability

Interviews with HCWs revealed that such shortages have a direct impact on patient behaviour, prompting some individuals to bypass primary care clinics and seek treatment at hospitals instead. The HCWs reported that patients who previously experienced medication stock-outs at clinics often chose to utilise hospital services for subsequent health concerns, including minor ailments, because of the perceived reliability of pharmaceutical availability in hospital settings:

‘Sometimes they complain about shortage of treatment at the clinic, so they decide to come to the hospital and avoid wasting time at the clinic, that’s what patients say.’ (Participant 6, Registered Nurse, Female)

‘Another thing that might be an issue in clinics is that you send the patients there and then they’ll come back and tell you that they were sent back because they don’t have sufficient or don’t have the drugs, some of the drugs that we do have in the hospital.’ (Participant 10, Medical Doctor, Female)

### Healthcare worker attitude

Interviews revealed that the demeanour of HCWs at PHC clinics played a pivotal role in shaping patients’ decisions to bypass these facilities in favour of hospitals. Several patients reported experiencing unprofessional conduct, including being shouted at, treated unfairly, or subjected to dismissive behaviour. These adverse experiences contributed to a perception of disrespect and lack of dignity within PHC clinic settings. Consequently, some patients opted to seek care at hospitals, where they reported receiving more respectful and compassionate treatment, even for minor health concerns:

‘They’re rude. They don’t treat us [*patients*] like human beings. It’s what I have heard from patients when they are here at the hospital.’ (Participant 3, Medical Doctor, Female)

‘They’ll tell you about the staff attitude in the clinics, they feel like they are mistreated there, and this pushes them to come to the hospital.’ (Participant 5, Operational Manager, Female)

The attitudes of some HCWs in PHC clinics were found to adversely affect the quality of care, particularly by inhibiting effective patient-provider communication. Interview findings indicated that some patients felt intimidated and uncomfortable when interacting with HCWs, which led to a reluctance to ask clarifying questions regarding their health or prescribed treatments. As a result, instances of incorrect medication usage were reported. Patients who later presented at hospitals often disclosed that they had received specific medications at the clinic but were unable to explain how they had been administering them. This lack of understanding was attributed to their hesitation to seek further guidance from clinic staff, stemming from prior negative experiences. Such communication barriers not only compromise treatment efficacy but also contribute to avoidable health complications:

‘What I have noticed is that our attitude as HCWs affect patients. I have had patients who have sought care at the clinic complaining about the attitude of HCWs. They sometimes end up not asking questions about how they should take medication, and they end up using it incorrectly and not seeing results. I think we really need to work on our attitude as this can really affect the patient’s health and wellbeing.’ (Participant 16, Professional Nurse, Male)

### Stigma, lack of privacy and confidentiality

The findings highlight that stigma influenced some patients’ decisions to bypass local clinics in favour of hospital-based care. The HCWs reported that patients deliberately avoided community clinics to prevent disclosure of their health conditions to neighbours, fearing social exclusion or differential treatment. This concern for privacy and potential community judgement led patients to seek care in hospitals, where they perceived a greater degree of anonymity and reduced risk of stigmatisation:

‘Some patients fear their neighbours [*fear of stigmatising comments*] so they decide to rather use hospitals than clinics because they can be easily seen at the clinic. I think they don’t want people from the community to know about their chronic diseases.’ (Participant 2, Operational Manager, Male)

‘Some of the challenges relate to clinics being located within the community. Some patients fear going to the clinic that is in the community where some people from the community work at that same clinic, patients can be stigmatised there.’ (Participant 3, Medical Doctor, Female)

In addition to stigma, concerns regarding confidentiality were identified as a factor influencing patients’ decision to bypass local clinics in favour of hospital-based care. The HCWs found that patients often preferred hospitals because these facilities are typically situated outside their immediate communities, thereby offering a greater sense of privacy. Patients expressed discomfort with seeking care at local clinics where their health conditions might be disclosed to neighbours, acquaintances, or community members. This concern was further compounded by the fact that some HCWs reside and work within the same communities as their patients, leading to fears that sensitive health information could be shared with family members or others in the community. Moreover, infrastructural limitations within clinics, such as inadequate private consultation spaces, were perceived to compromise confidentiality, reinforcing patients’ preference for hospitals where they felt their privacy was more secure:

‘Sometimes it is because of confidentiality where in their local clinics there are people they might know who works at the clinic, so they would rather move from there because there are sensitive issues that they would not want other people to know.’ (Participant 18, Enrolled Nurse, Male)

‘It all comes down to the confidentiality issue, If I were to be in my hometown, I would also go to another clinic rather than going to the one close to me because of confidentiality. Also in some clinics, the infrastructure limits confidentiality they don’t have enough consulting rooms, sometimes HCWs share rooms, so some patients go to the hospital because of this.’ (Participant 16, Professional Nurse, Male)

## Discussion

This study’s findings revealed that the majority of patients exhibited an accurate understanding of the healthcare referral system. Specifically, participants demonstrated awareness that PHC clinics are intended to function as the initial point of contact within the public health sector, in alignment with national referral protocols. In a study conducted in KwaZulu-Natal province, patients attending a district or regional hospital had knowledge of the referral system; however, this did not translate into appropriate service utilisation.^11^ Similarly, a study in the Free State province found that patients were aware of the referral protocol but chose to bypass PHC clinics because of concerns about service quality and accessibility.^28^ This aligns with the findings of qualitative interviews from this study, in which HCWs reported that patients, despite knowing the process, made decisions based on convenience, perceived quality, or dissatisfaction with clinic services. The presence of knowledge alone, without corresponding satisfaction with service delivery, appears insufficient to drive compliance with referral protocols. In the South African context, where patient knowledge is generally present, other contextual and systemic factors take precedence in influencing referral pathway adherence. The HCWs reported that patients frequently bypassed local clinics in favour of hospitals, citing long queues, limited consultation hours and most significantly, the absence of medical doctors at the clinic level. These perceptions fostered a belief among patients that better and more comprehensive care is only available at hospitals. These findings are aligned with a qualitative study of those seeking and delivering maternal health, TB and antiretroviral therapy services in South Africa, which showed that patients considered PHC facilities to be understaffed, under-resourced, and unable to manage even basic health concerns, prompting them to seek care directly from higher-level institutions.^29^ Similarly, in Kenya and Ghana, studies have shown that patients who perceive PHC services to be of low quality are more likely to bypass clinics, despite additional transport costs or longer waiting times at hospitals.^30^ These behaviours suggest that patients are not merely unaware of the referral process but are making rational decisions based on their prior experiences or expectations of care. This study found that shortages of essential medicines at PHC clinics contributed to patients bypassing clinics and seeking care directly at hospitals. The HCWs reported frequent stock-outs and incomplete treatment regimens as common challenges, which were perceived to undermine confidence in clinic-based services and influence care-seeking decisions. In a national survey led by the Stop Stock-outs Project, over 20% of surveyed clinics across multiple provinces reported being out of stock of one or more essential medicines at the time of the survey.^31^ A qualitative study conducted with 10 pharmacy managers in public sector hospitals in Gauteng province identified critical inefficiencies in procurement and supply chain systems as leading contributors to recurring medicine shortages in public hospitals and clinics.^32^ Similar issues were observed in Mpumalanga province, where delivery bottlenecks and poor forecasting from the Provincial Department of Health were cited as major barriers to consistent availability of essential medicines, further undermining PHC credibility.^33^

The HCWs acknowledged that patients often cited negative staff attitudes, such as being shouted at, rushed, or disregarded, as reasons for bypassing clinics. This trend is not unique to Mpumalanga province, as the study conducted in KwaZulu-Natal found that patients’ dissatisfaction with interpersonal conduct at PHC facilities led them to self-refer to district or regional hospitals, even for minor conditions.^34^ The implications of negative HCW attitudes are compounded by structural and systemic stressors. In under-resourced clinics, HCWs face heavy workloads, staffing shortages, and frequent interruptions because of supply or equipment issues; these are factors that may inadvertently affect their behaviour towards patients. Such pressures can result in burnout, emotional fatigue and poor communication, which patients interpret as poor service or disrespect.^35^ International comparisons affirm these dynamics. In studies across East and Southern Africa, patients identified trust and dignity as critical components of care quality, often prioritising respectful treatment over clinical infrastructure in their choice of facility.^36^ When PHC environments are perceived as uncaring or harsh, patients actively seek alternative care settings, even when these are further away, more expensive, or more crowded. The HCWs reported that patients often associated PHC proximity with social risk; they were fearful that community members or clinic staff might disclose their medical status or treat them differently. Consequently, patients often preferred to seek care from hospitals or facilities located outside their immediate communities, where they could preserve anonymity. The perceived risk of being seen at a local clinic, whether because of separate queues, file markings, or observable clinic flow, is a deterrent to PHC utilisation. Findings from a study in KwaZulu-Natal indicated that patients receiving HIV or TB treatment often preferred to access facilities far from their residence because of fear of gossip, stigmatisation, and discrimination within their community.^37^ The HCWs’ accounts in this study echoed the same sentiments, where patients from rural villages or farms bypassed nearby clinics to attend more distant hospitals to safeguard confidentiality. Beyond stigma, the structural set-up of many PHC facilities in South Africa can inadvertently compromise privacy. Facilities with inadequate consultation rooms, or where clinic workflows make it apparent what condition a patient is being treated for, risk violating patient confidentiality and discouraging continued use of services.^38^ This was reinforced in this study, where HCWs reported that patients actively avoided PHC services for fear of clinic staff who were connected to their social circles potentially revealing sensitive information. A 2022 report from the Human Sciences Research Council highlighted that even minor breaches of confidentiality, such as leaving patient records unattended or loud verbal interactions in open waiting areas, can erode trust in PHC services.^39^

### Limitations of the study

To achieve balanced geographic representation across all three districts in Mpumalanga province, hospitals were initially selected using routine DHIS data, with both main and back-up facilities identified. However, logistical constraints, particularly delays in obtaining site-level permissions, necessitated the inclusion of the first 10 hospitals that granted approval. The study was qualitative in nature, which means findings are often context-specific and based on small, non-random samples; this restricts the ability to generalise results to broader populations. Social desirability bias is another limitation that may have affected data accuracy. The HCWs may have been hesitant to disclose negative institutional practices because of concerns about professional repercussions. While confidentiality and voluntary participation were emphasised, such biases are inherent risks in facility-based studies involving sensitive service delivery themes. While the study provides important insights into patient behaviour and referral dynamics at the facility level, the findings are not generalisable to the wider Mpumalanga population. The sample was drawn exclusively from public-sector hospitals and therefore may not reflect the perspectives and experiences of individuals who exclusively utilise PHC clinics, private providers or traditional healers. The study focused on HCWs from selected public hospitals, but it could have broadened the understanding of bypassing if HCWs from PHC facilities had also been interviewed.

### Recommendations

Advocacy and community mobilisation efforts should be strengthened through Ward-Based Outreach Teams, community health workers, traditional leaders, and civil society organisations. These stakeholders should conduct structured community dialogues, household visits, and health education sessions to improve understanding of the referral system, including when and how patients should access PHC facilities before seeking hospital-based care. Clear, culturally appropriate messaging, delivered through community meetings, local radio, faith-based gatherings, and clinic open days, should emphasise the role of PHC as the first point of contact and clarify pathways for referral to higher levels of care. Mobilisation efforts should explicitly address stigma related to HIV, TB, and mental illness. This can be achieved by integrating anti-stigma messaging into routine health promotion activities, facilitating safe spaces for open discussion, engaging peer educators and community champions, and incorporating testimonies from individuals with lived experience. Strengthening confidentiality practices at clinics and improving respectful patient-provider communication should accompany these community-based interventions to build trust between communities and the health system. Improved health literacy and reduced stigma are expected to promote earlier care-seeking and more appropriate utilisation of PHC services. To address long waiting times and fragmented care, PHC facilities should implement structured appointment systems for stable chronic patients, including scheduled time slots, differentiated booking days, and reminder mechanisms (e.g. Short Message Service [SMS] or community health worker follow-up). In addition, facilities should promote integrated consultations for patients with comorbid conditions, whereby multiple chronic conditions are managed during a single visit by the same provider or through coordinated multidisciplinary care. These approaches can improve continuity of care, reduce unnecessary repeat visits, streamline patient flow, and ease congestion in facilities, thereby enhancing both operational efficiency and patient experience. Clinical support at the PHC level should be strengthened by expanding structured doctor outreach services to high-burden and remote clinics, with scheduled visits (e.g. monthly or bi-monthly) prioritising facilities with high referral rates or limited on-site clinical expertise. Outreach activities should include joint consultations, case discussions, and on-site skills transfer. These visits should be complemented by regular multidisciplinary case review meetings and ongoing clinical mentoring, either in person or through virtual platforms, to support nurses and other frontline providers in clinical decision-making. Strengthening on-site capacity in this manner may improve the quality of care, enhance provider confidence, and reduce avoidable referrals to higher levels of the health system. Staff performance and supervision should be strengthened through regular in-service training on respectful communication, confidentiality, and stigma reduction. These efforts should be reinforced by structured supervisory visits that monitor staff conduct, referral practices, queue management, and patient experience to promote accountability and continuous quality improvement. Medicine availability can be improved by implementing a DHIS-linked stock-monitoring dashboard to provide real-time visibility of stock levels, supported by buffer stock and emergency resupply mechanisms at facility and district levels. These measures are critical to preventing stock-outs, particularly for TB, HIV, non-communicable disease, and paediatric medicines, which are essential for continuity of care. Access to services should be expanded through extended operating hours, including pilot after-hours and weekend services in high-volume peri-urban and semi-rural facilities. The success of the pilot should be evaluated using indicators such as appointment adherence, service utilisation, and patient satisfaction. This strategy would improve access for working patients and those unable to attend clinics during standard operating hours, thereby reducing missed appointments and treatment interruptions. Finally, hospital gatekeeping should be strengthened through the enforcement of standardised triage protocols and mandatory documentation for non-urgent walk-in patients, with hospital management conducting periodic compliance monitoring. Improved communication between clinics and hospitals, supported by referral registers and digital tools, will close feedback gaps, enhance coordination of care, and promote efficient use of health system resources.

## Conclusion

This study provides an analysis of the underlying factors contributing to the bypassing of the referral system in Mpumalanga province. Several interrelated factors were identified as key drivers, including long waiting times, medicine stock-outs, perceptions of poor quality at PHC facilities, lack of trust in HCWs, and concerns around privacy and confidentiality. These findings suggest that patients make healthcare-seeking decisions based not only on medical needs but also on previous experiences, structural limitations, and the perceived responsiveness of the health system. The cumulative effect of these behaviours contributes to hospital overcrowding, strains limited resources and undermines the efficiency and equity of the province’s tiered health service delivery model. The evidence presented in this study underscores the urgent need to strengthen the performance, accessibility, and public trust in PHC services in order to restore the intended functionality of the referral system and improve overall system resilience.
